# Experimental Medicine, &c.

**Published:** 1868

**Authors:** L. Ranvier

**Affiliations:** Paris


### ARTICLE VI.

*Experimental Medicine—Experimental Researches into the Subject of the Action of Phosphorus upon Living Tissues —Reflections upon the Pathogenesis of Fatty Transformations.* By Dr. L. Ranvier, Paris. Translated expressly for the Journal, by Walter Hay, M. D., Chicago.

### SECOND SERIES OF EXPERIMENTS.

*Experiments* 4, 5, *and* 6.—On the 15th of October I placed similar bits of phosphorus under the skin of the loins of two other frogs; I killed one of them on the 23d and the other on the 28th. In neither of them were any inflammatory phenomena in the vicinity of the phosphorus. In the first there were observed yellowish plates and striæ upon the surface and in the interior of the liver; at these points the hepatic cells were loaded with fatty granulations. In the second the liver was completely degenerated.

A sixth frog was poisoned by the very same process on the first of December, 1866, and exhibited to the Society on the 15th of the same month. It presented no inflammatory phenomena in the neighborhood of the foreign body, and the fatty transformation of the liver and kidneys was very complete.

In order to establish the value of these experiments, it was necessary to insert under the skin of different frogs fragments of inert substance, and to ascertain if they determined around themselves congestions, exudations, or neoplasmata. This I accomplished in a sufficiently large number of animals, and ascertained that the presence of an inert foreign body, such as a little pebble or a fragment of thread placed in the lumbar region of the frogs, produced very speedily congestion, exudation, and even hyperplasia of the conjunctive tissue of the aponeurotic envelope, and of that which accompanies the cutaneous nerves, to such a degree that at the end of three or four days the foreign body is completely enveloped by a mass of new formation. Moreover, a variable quantity of serosity accumulated under the skin. None of these phenomena, as has been seen, are produced around a fragment of phosphorus.

*Experiment* 7.—On the 24th of September, 1866, I introduced, by means of a sub-cutaneous incision between the ears of a young rabit, a fragment of phosphorus of seven millimetres in length, by two in width and thickness. Next I removed the os calcis of a new-born rabbit, and placed it in the right flank of the first rabbit. On the following days no inflammatory phenomena supervened on the side of the phosphorus, whilst at the point where I placed the os calcis I determined the presence of puffiness and tenderness upon pressure. Matters remained in this state until the 4th of October. At this date I killed the animal, and ascertained that the engrafted os calcis was surrounded by a thick, whitish deposit of two to three millimetres in extent, formed of embryonic cells. Sanguinous vessels ramified already in this deposit, which later, as I have determined by other experiments, would have given origin to more perfect tissue. In the vicinity of the phosphorous nothing similar was recognized; moreover, the phosphorus had preserved its transparency, and its volume was not diminished by an appreciable quantity, whilst the connective tissue circumjacent did not appear to have undergone any modification; it was not hyperæmic, nor infiltrated by exudation. Examined with the microscope, it appears with its fundamental fibrillated substance. These cells,' at some points, were more developed than is usual, but there was no very evident proliferation. The liver, the kidneys, the muscles, had not undergone fatty degeneration, which was probably due to the effect that the toxic substance had not been absorbed in sufficient quantity.

In order to give its entire value to this experiment, I should add that I introduced into the sub-cutaneous cellular tissue of different rabbits, inert foreign bodies, and constantly established, at the end of a few days, suppurative inflammations.

And if, in this experiment, I introduced under the skin of the animal on one side a fragment of phosphorus, and on the other a portion of living tissue, I did so in order to give the greatest possible value to the experiment, since an animal graft made with care rarely entails suppurative inflammation.

*Experiment* 8.—The 10th of December a fragment of phosphorus was placed under the scalp of a Guinea pig, the hair of the region having been carefully cut away.

On the following days no swelling was established, and pressure determined no pain. This condition of affairs existed up to the 24th of December; on that day the animal died accidentally. In the vicinity of the phosphorus there was neither hyperæmia nor exudation; however, the surrounding connective tissue had undergone a slight thickening, and it was determined by the microscope that the number of the cells was notably increased. The different organs presented no alteration.

In this case, in the vicinity of the phosphorus, slight evidences of irritation were observed, but they were very much less than those determined by the pressure of inert bodies.

In these different experimens, fragments of phosphorus and of other foreign matter were placed simultaneously in the same animal, or in different animals of the same species. In none of these experiments did phosphorus, in its pure state, deposited in the midst of the tissues, and out of contact with the external air, determine around itself any such inflammatory phenomena as those produced by inert matters. Whilst a fragment of phosphorus introduced into living tissue represents, by its persistent angular form, and by its consistence a veritable foreign substance; it should, like it, determine inflammatory phenomena, if its action as a foreign substance were not counterbalanced by its specific action. This action, which removes from the cells, at least in part, the property of undergoing formative irritation, should therefore be considered as contra-stimulant. It can, therefore, to-day be no longer admitted that the fatty transformations which supervene in the liver, the kidneys, the muscles, etc., under the influence of phosphorus, are due to the irritant action of this substance. It becomes necessary to resort to other explanations.

There are met with in science three other theories: that of G. Lewin,* which consists in the admission that phosphorus introduced into the digestive canal suppresses entirely the absorption of fat by the chyliferous vessels. The veins would act vicariously for them, and the chyle would penetrate the vena porta, and be carried directly to the liver. The cells of this organ, brought into contact with a blood loaded with fat, would become infiltrated therewith. This theory rests upon exact facts.

Indeed, animals to whom phosphoretted oil has been administered, killed from three to four hours after the injection of the toxic substance, have their chyliferous vessels filled with a serous liquid, whilst the portal vein contains blood loaded with fine fatty granulations. It is possible even to administer to the animals ether with the phosphoretted oil without diminishing the transparency of the chyliferous vessels. This experiment, which I have reproduced many times, demonstrates that the phosphorus has a powerful action in preventing absorption by the lymphatics of the intestine; for, as M. Claude Bernard† has taught us, ether has the remarkable property of stimulating the absorption of fats by the chyliferous vessels.

* Lewin, Etudes sur l'empoisonnement par le Phosphore, Arch. de Virchow, 2d Serie, t. 1, 1861.

† Cl. Bernard, Lecors sur les effets des matoeres toxiques et medicamentences, 1857.

This theory of G. Lewin would be satisfactory if the phosphorus introduced into the digestive canal determined fatty transformations in the liver alone; it was formulated by its author at an epoch when it was not known that the fatty transformation involved a great number of organs, might be generalized for all the lymphatics the idea of Lewin concerning the action of phosphorus upon the lymphatics of the intestine, and maintain that if phosphorus determines fatty transformation in different organs, it is because the lymphatics, having for their function the resorption of the fat which they elaborated, physiologically, are impeded in this function. But, as will be perceived, this would be a substitution for the hypothesis of Lewin of another hypothesis, in support of which not one fact exists.

The second theory is that of Munk* and Leyden. These two authors having observed that different inorganic acids and certain substances, such as arsenic and antimony, as well as phosphorus, determine fatty transformations, (polyorganic) were impressed with the idea that these toxic statoses were the result of the destruction of the red globules of the blood.

In order to demonstrate the inaccuracy of their mode of observation, it suffices to poison frogs with phosphorus, and then, whilst they are still living, to study their circulation by the aid of the microscope. It can thus be established that the red globules which circulate in the capillaries of these animals have undergone no modification in their color or in their form. I have repeated this experiment several times, and it has always given me negative results. Moreover, with M. Demonchy we have determined that in frogs poisoned with Tartar emetic or arsenious acid, the blood had sustained no morphological alteration; and yet the liver and kidneys of these frogs were in a state of complete fatty transformation.

I have frequently examined the blood of rabbits and cats poisoned by phosphorus, and have never been able to distinguish any alteration of the red globules which could be attributed to the poisoning.

We now come to a third theory, consisting in the claim that phosphorus determines fatty transformations by reason

---

* Munk and Leyden, Dir aerzte phosphovergiftung, etc. (Rucksuht auf. Path. u. Phys., 1865.

of a specific property. Upon the side of this prudent reservation M. Larcereaux* ranges himself.

In truth, in the present state of science it is difficult to explain the transformations which supervene upon poisoning by phosphorus; but what strikes our attention is this: that amongst toxic substances phosphorus is not the only one which determines fatty degenerescence. It should not, therefore, be claimed for it that it possesses a specific action, and hence one is induced, by the example of Munk and Leyden, to seek the relations between phosphoric steatoses and other fatty degenerescencies.

This brings me to the second part of this work: to the pathogenesis of fatty transformations, and especially to their relations with the inflammatory process.

We have seen Virchow maintain that inflammation can extend itself into the muscles and into different parenchymata by means of a fatty degenerescence of the histological elements.

In a final stage of neo-formations, inflammatory or otherwise, a fatty transformation of the cells supervenes habitually. Does it follow that this transformation appertains to a developmental movement which characterizes inflammation? Assuredly not. It is seen also in the numerous experiments of Virchow that fatty degenerescence is a process essentially passive. It is then incomprehensible how this illustrious professor can maintain that in certain cases inflammation, a phenomenon essentially active, could be characterized by fatty transformations.

Facts sufficiently numerous show, moreover, that inflammation and fatty degenerations, in place of being connected in an intimate manner, are, on the contrary, in opposition. In the phlegmon of sub-cutaneous cellular tissue, the adipose cells lose the fat which they contain, their nuclei and the little mass of protoplasma which surrounds them, originate by division to a very abundant production of cells, which fill up the old adipose vesicle. In acute osteitis the adipose medullary is seen to transform itself into embryonic medulla by an identical mechanism.† In acute or chronic arthritis, the cartilaginous cells, which physiologically contain fat, are deprived of it during the time whilst the cellu-

* Loc. cit.

† Des alterations des cartilages dans les tumeurs blanches. (Bull. de la Soc. Anatom., 1865.)

lar proliferation persists. This disappearance of the fat in the elements which contain it in a phosiological state is met with not only in the inflammatory process, but also in all the active neo-formations. Then, when the neo-plasmata which constitute tumors take their point of departure in the cellular-adipose tissue, or in the medulla of bone, they determine the disappearance of the fat in the cells which they involve.

But a still more important fact: when under the influence of a pathological cause, the fatty transformation has invaded certain cells, it is seen that these cells can rid themselves of the fat which they contain under the influence of inflammation, if at any time it should suvervene before the cellular elements may have been completely destroyed by degeneration, as results from investigations which I have made into the alterations of diarthrodial cartilages in white swellings.*

However, at the end of the inflammatory process, and in the last phase of every neo-formation, a fatty transformation of the elements, at that time superabundant, is observed. This transformation should not be attributed to an imitative process, but entirely to an alteration of nutrition, for it never supervenes at the moment when the cells are in full proliferation. It happens only at the time that the formative movement is arrested, and that the nutritive exchanges become difficult for the elements whose number is no longer in relation with the vascular development. On the first day of a catarrhal inflammation, the exudation is transparent, and the numerous cells which it contains show themselves with every indication of a very active multiplication, and do not contain a single fatty granulation. Later, when the exudation becomes yellowish and opaque, nearly all the cells enclose granulations, and even some little drops of fat.

It will be remembered that in the month of September frogs accumulate fat in their muscles. This fat is probably destined to nourish the animal during hybernation—it is known, indeed, that the frog has no sub-cutaneous adipose cellular tissue—it accumulates fat in the great epiploon and in the muscles. It was interesting to see whether an irritation directed to the fatty muscles of the frog could diminish or remove the fatty granulations—granulations arranged

* L. Ranvier, Considiration sur le developpement du tissu osseux, etc., 1865.

like beads between the elementary fibrillæ. To determine this result, I passed threads through the muscular masses. I effected fractures, and established the fact that from the fifth to the eighth day the fatty granulations had considerably diminished with having completely disappeared in the portion submitted to the irritation. It is probable that this result would be more or less rapid, according to the season of the year, and according to the temperature. My experiments were conducted upon six frogs; four have had the femur fractured; three only were killed from the sixth to the eighth day. In the cases of two of those which had sustained fractures, I awaited the twentieth day, until there had been a commencement of a callus; in these, a limited number of muscular fibres were comprised in the cartilaginous mass, and had undergone complete fatty degeneration. In the last, which was examined twelve days after the experiment, the muscular bundles in the neighborhood of the thread, upon whose passage there had been an abundant cellular neo-formation, had commenced also to undergo fatty degeneration; in these last cases, the fatty transformation ought not to be interpreted by the inflammation, but very much rather to the hindrance sustained in the nutrition of the muscular fibres by the presence of tissues of neo-formation between these fasciculi.

I come now to the part the most disputed, and in fact the most difficult of fatty transformations. Whence comes the fat which infiltrates the histological elements? Is it already formed in the blood which merely deposits it in the cells? Does it originate, as there is much evidence to show, from a direct transformation of the albuminoid substance which forms the protoplasma of the cells? Is its accumulation in the cells, the result of a deposit or of an exaggerated formation, or, indeed, is not the fat formed physiologically, consumed just in proportion as it is produced? Finally, it may even be asserted that the fatty matters contained in the cells are taken up, little by little, by absorption, and an impediment applied to this function would thence determine a fatty accumulation. It is not my intention to reply to these different questions which have been already disposed of partially, and very imperfectly resolved by Wagner, Mideldorf, Witich, Virchow, etc.; but I desire at present simply to adduce some new evidence. The microscope does not always suffice to detect fat contained in organic liquids or histological elements. Thus in the blood no fatty granulations are recognized, whilst it contains fat in notable quantity. Fatty matters enter into the composition of the red globules in the proportion of 18 to 25 per cent.,* and yet no fatty granulations are to be distinguished in the globules.

These fatty matters, which can not be discovered by the microscope, as is very well established for the red globules of the blood—very probably because they are combined in an intimate manner with other constituent matters, can resume their form and characteristic reaction under certain conditions; when the blood has escaped from the vessel, and remains in an accidental receptacle, it is observed that the red globules become decolorized, abandoning their hæmatosine, which is dissolved in the surrounding liquids or concretes in the form of granulations or crystals; moreover, these globules become spherical, lose in the direction of their larger diameter, which falls to five ten thousandths of a millimetre; then is perceived, forming themselves under a membrane which seems to envelop them, some fatty granulations disposed like a string of beads: these granulations are insoluble in acetic acid, and have all the optical characteristics of fat.

In this case I would say that the fat was masked in the globule, and that it had become apparent at the end of chemical transformations as yet badly defined. I will not maintain, with Försher, that a protein substance is transformed into fat.

This theory of fat masked can explain many facts; it is in relation with certain very interesting phenomena which occur in the bodies of fœti which, after having died, have remained from one to three weeks in the uterus.

During the past year I have been able to collect five of these fœti, which I have studied carefully.

In regard to the question which occupies us now, they presented identical modifications, which will obviate the necessity of making special observations upon each one of them.

The blood contained fatty granulations; in four of them the red globules were entirely destroyed; in the fifth a few were recognizable. In the nerve tubes the myelene was fragmentary, and one would have said that the nerves had

* Pelonze et Fremy, Traite de Chimie, generale, tome VI., 2d edition, p. 100.

degenerated after section. In the nervous centres nothing more was found than a semi-fluid mass of fatty granulations, crystals of fat, and cholesterine. The nervous cells alone were intact, and contained no fatty granulations. The liver contained great quantities of fat, and the hepatic cells were destroyed: the epithelial cells of the renal tubuli contained very distinct fatty granulations. The cartilaginous cells of the ossific layer, which in the physiological state contain no fat, apparently, at this period of life, contained from one to five fatty granulations. The primitive fasciculi of the muscles of the trunk and of the limbs, the fundamental substance of the cartilages, the cells of the connective tissue, the fundamental substance of this tissue, contained in no case fatty granulations. On the other hand, the muscular fasciculi of the heart were all loaded with it.

From these facts it must be concluded that albuminoid substances do not give origin to the formation of fat when they are abandoned to themselves—that is to say, when they are deprived of life—for if it were not so, it would be impossible to comprehend how the substance of the muscles of the limbs, placed under the same conditions as the muscles of the heart, does not give origin to the formation of fat, whilst in these last, fatty granulations appear. Moreover, the formation of fatty granulations in the cases which we are now discussing, is limited actually for each histological element. Some few granulations only show themselves in the cells of the cartilages; they are a little more abundant in the cells of the kidney; they are out of all proportion in the liver; and, finally, in the nervous system, they seem to originate directly from the medullary matter; for the fibres of Remak, so abundant in the fœtus, contain here no fatty granulations. I perceive but one mode of explaining these different facts: it is by the assumption that the fatty matters combined more or less feebly with the albumenoid matters are more or less abundant according to the different histological elements. These fatty matters are masked in the different tissues whilst living, and disengage themselves, little by little, after death. It is easy to perceive how interesting would be the results of chemical investigations made in this discretion, but I am not yet in a position to give positive results upon this subject. We have, however, only an hypothesis based upon certain facts, and which I hope to demonstrate completely in another work.

It is now apparent why certain elements involved in necrosis might give origin to fatty granulations, without necessitating the intervention of a direct transformation of albuminoid matters into fat. But these fatty granulations would be in this case, limited in number; as in the conditions in which much fat originates in the cells. It is necessary, therefore, to seek the aid of a deposit more considerable, a greater elaboration, in default of assimilation or absorption.

If we recur now to the interpretation of fatty transformations in poisoning by phosphorus, resting upon the experiments contained in this memoir, experiments which demonstrate that phosphorus impedes nutrition and the multiplication of cellular elements, to such a degree that the phenomena of inflammation can no more develop themselves, we will understand how these elements can no longer elaborate the fat which they contain in a masked condition, or that which is brought to them by the vascular system.

What corroborates greatly this view of the matter, is that in poisoning by phosphorus, the first organs involved in the fatty degeneration are the liver, the kidneys, and the heart, organs in which fat is recognized in the fœtus which has remained dead for some weeks in the cavity of the womb.

CONCLUSIONS.

The protoplasma of the cells appears to be the seat of the exchanges, and of the elaboration of the material carried by the blood; it is likewise in the protoplasma that the fat is first deposited.

The presence of fat in a cell which contains none of it apparent, in a normal state originates from the fact that the nutritive process in the cell is retarded. If this movement is stimulated by irritation, the fat disappears.

Since certain cells manifest great activity in the elaboration of fat, it does not result from this that they form it at the expense of available substances which traverse them; for the blood contains fat in a masked condition—that is to say, in a state of combination or of saponification. The adipose cells, those of the liver, for example, appear to reduce the combined fat to a neutral or insoluble condition.

In the case of the fœtus, dead, and remaining for some time in this condition in the uterine cavity, the masked fat becomes apparent to the microscope in the liver, the kidneys, the heart, and the cartilage cells.

Phosphorus determines fatty transformations because it diminishes the nutrition of the histological elements, because it is a contra-stimulant to these elements, a property demonstrated by the experiments detailed in this manner.*

